# Measured and modelled effect of land‐use change from temperate grassland to *Miscanthus* on soil carbon stocks after 12 years

**DOI:** 10.1111/gcbb.12624

**Published:** 2019-05-21

**Authors:** Amanda J. Holder, John Clifton‐Brown, Rebecca Rowe, Paul Robson, Dafydd Elias, Marta Dondini, Niall P. McNamara, Iain S. Donnison, Jon P. McCalmont

**Affiliations:** ^1^ Institute of Biological, Environmental and Rural Sciences (IBERS) Aberystwyth University Aberystwyth United Kingdom; ^2^ Centre for Ecology & Hydrology, Lancaster Environment Centre Bailrigg, Lancaster United Kingdom; ^3^ Institute of Biological and Environmental Sciences University of Aberdeen Aberdeen United Kingdom; ^4^ College of Life and Environmental Sciences University of Exeter Exeter United Kingdom

**Keywords:** bioenergy, land-use change, life cycle assessment, *Miscanthus*, pasture, soil organic carbon

## Abstract

Soil organic carbon (SOC) is an important carbon pool susceptible to land‐use change (LUC). There are concerns that converting grasslands into the C_4_ bioenergy crop *Miscanthus* (to meet demands for renewable energy) could negatively impact SOC, resulting in reductions of greenhouse gas mitigation benefits gained from using *Miscanthus* as a fuel. This work addresses these concerns by sampling soils (0–30 cm) from a site 12 years (T_12_) after conversion from marginal agricultural grassland into *Miscanthus x giganteus* and four other novel *Miscanthus* hybrids. Soil samples were analysed for changes in below‐ground biomass, SOC and *Miscanthus* contribution to SOC (using a ^13^C natural abundance approach). Findings are compared to ECOSSE soil carbon model results (run for a LUC from grassland to *Miscanthus* scenario and continued grassland counterfactual), and wider implications are considered in the context of life cycle assessments based on the heating value of the dry matter (DM) feedstock. The mean T_12_ SOC stock at the site was 8 (±1 standard error) Mg C/ha lower than baseline time zero stocks (T_0_), with assessment of the five individual hybrids showing that while all had lower SOC stock than at T_0_ the difference was only significant for a single hybrid. Over the longer term, new *Miscanthus* C_4_ carbon replaces pre‐existing C_3_ carbon, though not at a high enough rate to completely offset losses by the end of year 12. At the end of simulated crop lifetime (15 years), the difference in SOC stocks between the two scenarios was 4 Mg C/ha (5 g CO_2_‐eq/MJ). Including modelled LUC‐induced SOC loss, along with carbon costs relating to soil nitrous oxide emissions, doubled the greenhouse gas intensity of *Miscanthus* to give a total global warming potential of 10 g CO_2_‐eq/MJ (180 kg CO_2_‐eq/Mg DM).

## 1 INTRODUCTION

Energy generation from fossil fuels (e.g. coal and gas) must be phased out as part of world‐wide efforts to combat the impacts of climate change (IPCC, 2014). The European Union has set a target for renewable energy (wind, solar, hydro and bioenergy) to reach a minimum of a 27% share of the energy generation mix by 2030 ([Link]) from the current share of ~17% (European Commission, 2017). In the United Kingdom, renewable energy other than wind, solar and hydro accounted for 9.4% of the total energy produced in 2017 and there is scope for bioenergy generation (e.g. from biomass crops, landfill, and sewage gas and anaerobic digestion) to increase (BEIS, 2018).

Agricultural grasslands represent a third of the utilized agricultural area across Europe (Eurostat, 2018) and due to changes in farming subsidies and temperate grassland agricultural management across Europe, areas of lower grade agricultural grassland may become available for biomass crops (Donnison & Fraser, 2016; Taube, Gierus, Hermann, Loges, & Schönbach, 2014). In the United Kingdom, Welsh agriculture is primarily grass based (Welsh Government, 2018) and spatial modelling has suggested that there may be 0.5 M ha suitable for the planting of perennial bioenergy crops (such as *Miscanthus* and short rotation coppice; Lovett, Sünnenberg, & Dockerty, 2014). However, there are concerns that losses of soil carbon (C) caused by soil disturbance (Balesdent, Chenu, & Balabane, 2000; Conant, Easter, Paustian, Swan, & Williams, 2007) could reduce the C mitigation benefits gained from the conversion of grasslands into the production of bioenergy crops (McCalmont, Hastings, et al., 2017; Whitaker et al., 2018).

The biomass crop* Miscanthus x giganteus* (*Mxg*; Greef & Deuter, 1993) is a commercially available hybrid that is a fast‐growing, tall perennial grass, with an efficient C_4_ photosynthetic pathway. It is a low‐input crop with the potential to be grown on agriculturally marginal land (Clifton‐Brown, Schwarz, & Hastings, 2015; Lewandowski, Clifton‐Brown, Scurlock, & Huisman, 2000). Compared to annual crops, *Miscanthus* has the potential to sequester C due to reduced soil disturbance (tillage is only required as part of the initial cultivation; Post & Kwon, 2000), the translocation of C from above‐ground biomass to roots and rhizomes (Kuzyakov & Domanski, 2000), and the provision of soil C inputs from leaf litter (Amougou, Bertrand, Machet, & Recous, 2011). New, commercially relevant *Miscanthus* hybrids are being developed with different morphologies and traits (Lewandowski et al., 2016; Nunn et al., 2017) which may impact soil organic carbon (SOC), for example though variations in leaf litter and carbon allocation between above‐ and below‐ground biomass (Clifton‐Brown & Lewandowski, 2000; Richter, Agostini, Redmile‐Gordon, White, & Goulding, 2015).

Land‐use change from arable crop production to *Miscanthus* generally shows an increase or no change in SOC, whereas, in contrast, it has been found that *Miscanthus* plantations have lower or similar SOC when compared to grassland controls (Qin, Dunn, Kwon, Mueller, & Wander, 2016). However, to date, most studies have taken grassland sites adjacent to *Miscanthus* plantations as representative of pre‐cultivation conditions (Clifton‐Brown, Breuer, & Jones, 2007; Foereid, Neergaard, & Høgh‐Jensen, 2004; Rowe et al., 2016; Schneckenberger & Kuzyakov, 2007; Zang et al., 2018; Zimmermann, Dauber, & Jones, 2012), and while the use of such sites where soil and climate conditions are similar can provide a reasonable indication they may not accurately replicate baseline SOC stocks (McCalmont, Hastings, et al., 2017; Richter et al., 2015). Therefore, there is a need to reduce some of the uncertainty around the impact of this LUC from grassland to *Miscanthus* on SOC (Whitaker et al., 2018), especially over the longer term.

Any carbon losses or gains from LUC should be considered over the expected lifespan of the *Miscanthus* crop, currently estimated to be between 10 and 15 years **(**Clifton‐Brown et al., 2015). Clifton‐Brown et al. (2007) found an increase in SOC under 15 year old *Miscanthus* compared to an adjacent grassland, whereas Zang et al. (2018) found that although SOC increased between samples taken at the same site 9 and 21 years after conversion, SOC was similar to samples taken from a neighbouring grassland (used to represent pre‐conversion conditions). Reducing the uncertainty around the long‐term impact of SOC using pre‐cultivation data from the same site is needed to inform soil carbon model predictions and life cycle analyses (LCA).

Due to the limited number of long‐term empirical studies of land use conversion into energy crops, a number of models have been used to estimate changes in SOC (Robertson, Davies, Smith, Dondini, & McNamara, 2015). ECOSSE (Estimation of Carbon in Organic Soils: Sequestration and Emissions) is a process‐based model that has been successfully tested and used for simulating SOC under perennial energy crops including grassland and *Miscanthus* in this UK region (Dondini et al., 2015; Dondini, Richards,Pogson, Jones, et al., 2016; Dondini, Richards, Pogson, McCalmont, et al., 2016b). However, empirical baseline data of SOC stocks in LUC from grassland to *Miscanthus,* coupled with data of SOC stocks under the mature crop (over 10 years old) would provide further model validation. ECOSSE can be used at the site or regional scale and represents an improvement on a previous model, RothC, due to a new approach to mineral and organic soils whereby the extent of processes occurring are adjusted according to soil conditions and not differentiated solely by soil type (Robertson et al., 2015; Smith et al., 2010).

LCA is a tool that can provide an indication of the environmental costs or benefits of producing energy from different methods and by enabling comparisons which help to inform policy decisions relating to proposed LUCs (McManus & Taylor, 2015). LCA's relating to LUC from grassland to *Miscanthus* have not included changes in soil carbon due to a lack of reliable data, and have tended to assume no change or an increase in SOC stocks (Hastings et al., 2017; Hillier et al., 2009). LCA estimates involving LUC are sensitive to the initial land use and condition (McManus & Taylor, 2015). For example, Robertson et al. (2017) investigated SOC as part of their LCA involving LUC to *Miscanthus* but this was from a previous arable land use with annual cultivation; potential losses at grassland sites, with less regular soil disturbance, could have a significant impact on LCA results (Hillier et al., 2009). Changes in SOC over the lifetime of the crop also have the potential to impact on greenhouse gas balances to a greater extent than other LUC associated costs such as increased soil nitrous oxide (N_2_O) emissions (Whitaker et al., 2018).

Therefore, in this study, we aimed to (a) measure the change in SOC stock, and *Miscanthus* contribution to SOC, from a mature (>10 years old) *Miscanthus* crop following land use conversion from an agricultural grassland compared to baseline data of initial SOC stocks; (b) use the empirical data obtained to provide validation for ECOSSE model predictions; (c) use the ECOSSE model to predict SOC stocks following LUC from grassland for an estimated *Miscanthus* crop commercial lifetime of 15 years along with a continued grassland counterfactual, in order to establish the difference in SOC between the two scenarios; and (d) provide context for the predicted difference in SOC between the *Miscanthus* and grassland scenarios at the end of the 15 years by converting the difference in a global warming potential (GWP) for inclusion in an LCA comparison per unit of energy based on the heating value of the *Miscanthus* biomass.

In order to achieve this, we built on previous experimental work reported in Zatta, Clifton‐Brown, Robson, Hastings, and Monti (2014) which although from a single site includes baseline SOC data (T_0_) and data taken from the same site 6 years (T_6_) after land use conversion from grassland into *Mxg* and four novel *Miscanthus* hybrids. Taking advantage of the difference in δ^13^C natural abundance values arising from the contrasting C_3_ photosynthetic pathway of temperate grassland species compared to the C_4_ pathway of *Miscanthus* (Kuzyakov & Domanski, 2000), we assessed changes in the contribution of *Miscanthus* to SOC between T_6_ and T_12_.

## 2 MATERIALS AND METHODS

Sampling was conducted at a replicated plot trial situated at Aberystwyth, Wales, UK (52°26′ N, 4°01′W) on agriculturally marginal shallow dystric cambisol and dystric gleysol classified soil (up to 0.6 m soil depth in places but mainly with a gravel layer at depths >0.3 m). Prior to conversion, the site was a mature established perennial ryegrass sward. Historically, the site has predominantly been used for grass pasture and silage trials (resown ~5 yearly) with occasional oat crops (Zatta et al., 2014). The sample area consisted of four blocks of five randomized 25 m^2^ plots; each plot contained one of five different *Miscanthus* hybrids. In September 2004, prior to planting, the existing mature perennial ryegrass sward was sprayed with glyphosate (3 L/ha) and inversion tilled with mouldboard plough and power harrow before a ryegrass cover crop was sown in October 2004. The cover crop was sprayed with atrazine (3 L/ha) on 5 April 2005 with the *Miscanthus* planted on 24 May 2005.

### 2.1 *Miscanthus* hybrids

Bare root transplants of four novel hybrids (*M. sacchariflorus x M. sinensis*) cloned via in vitro tillering (hereafter Hyb 1, Hyb 2, Hyb 3 and Hyb 4), and rhizome segments of the commercially available *Mxg* were slot planted at a density of two plants per square meter. Compared to *Mxg*, after 3 years growth, Hyb 1–4 had a higher stem density (~39 vs. 30 stems m^2^), lower canopy height (~2.05 vs. ~2.50 m) and lower above‐ground biomass lignin (~10% vs. ~30%; P. Robson & J. Clifton‐Brown, unpublished data).

The hybrids formed part of an ongoing yield trial with data recorded each year. Percentage differences between the above‐ground autumn peak harvest and spring harvest (ripening loss) for each hybrid were calculated from the oven‐dried weights of 10 stems taken from each plot in November 2007 and February 2008.

### 2.2 Soil cores

Detailed methods regarding the pre‐planting (6 May 2005, T_0_) soil cores and those taken after 6 years of crop growth (5 May 2011, T_6_) can be found in Zatta et al. (2014). Briefly, at T_0_, five core samples (to 30 cm depth) were taken from two plots in each block, and at T_6_, three core samples were taken from each plot. Each of the three T_6_ core locations was taken to represent a portion of the overall field area covered by plant centre (8.1%), plant edge (24.5%) and inter‐row (67.4%).

On 4 and 5 May 2017, 12 years since the plots were planted (T_12_), three cores were again taken in each plot following the methods at T_6_. The same 8.5 cm diameter cylinder auger (Eijkelkamp, Giesbeek, The Netherlands) was used with a Cobra TT jackhammer (Atlas Copco, Hemel Hempstead, UK) to take intact and uncompressed cores at three locations in each plot taken to represent a percentage of the overall field area. The soil core locations, individual plot heterogeneity and details of the field cover survey used to calculate the percentage area represented by each core are given in the Supplementary Information (S1 & S2). At T_12_, the area represented by the plant centre (C_c_) was determined to be 9.82%, the plant edge (C_e_) 53.39% and the inter‐row (C_i_) 36.79%. Soil cores were taken to a depth of 30 cm at position C_i_, 31 cm at C_e_ and 32 cm at C_c_ to allow for soil displacement by rhizome growth (Zatta et al., 2014) and were subsequently split at 15 cm, 16 cm and 17 cm, respectively, before air drying to a constant weight. Soils were sieved (2 mm) to separate soil, stone and below‐ground biomass (roots and rhizome). Soil was then ball milled (Planetary Mill, Fritsch GmbH, Idar‐Oberstein, Germany). Air‐dried below‐ground biomass (roots and rhizome) were premilled (SM100, Retsch GmbH, Haan, Germany) before being finely cryomilled (6,870 Cryomill, SPEX, Stan‐hope, UK) in liquid nitrogen. Bulk density was calculated using the same method as described in Zatta et al. (2014).

### 2.3 Carbon analysis

Inorganic carbon was removed from a 3 g portion of each milled soil sample by adding 30 ml 1 M HCl, rinsing and oven drying to constant weight at 40°C (Clifton‐Brown et al., 2007). A quantity of 200 mg of the acid‐treated soil was analysed for percentage carbon content by combustion using a Vario Macro Cube (Elementar Analysensysteme GmbH, Langenselbold, Germany). Total organic carbon was calculated using Equation (1):

(1) $$\text{SOC}=\text{POC}\times \left({\text{ODW}}_{\text{acid}}/{\text{ODW}}_{\text{initial}}\right)$$

where SOC (%) is the total soil organic carbon, POC is the percentage organic carbon in the acid‐washed sample, ODW_acid_ is the oven‐dried weight of the sample after acid washing and ODW_initial_ is the oven‐dried weight of the same sample before acid washing.

SOC mass was calculated in two ways: to a fixed soil depth (using the soil bulk density) and to an equivalent soil mass (ESM; Ellert & Bettany, 1995; Wendt & Hauser, 2013). For the ESM approach, Equations (2) and (3) were used with a fitted cubic spline curve (Wendt & Hauser, 2013) to provide estimates of the cumulative ESM for a layer of soil mass 0–3,000 Mg/ha (SOC_ESM_). The SOC mass for both methods was then scaled up to Mg/ha using the percentages relating to the representative area covered by each core location.

(2) $${\text{M}}_{\text{soil(DL)}}=\left({\text{M}}_{\text{sample}}/{\text{A}}_{\text{sample}}\right)\times 10^{4}$$

where M_soil(DL)_ is the mass of soil in the depth layer (Mg/ha), M_sample_ is the dried mass of the soil core sample (g), A_sample_ is the area of the core sample (mm^2^) and 10^4^ is the conversion factor from g/mm^2^ to Mg/ha.

(3) $${\text{SOC}}_{\text{ESM}}=\left({\text{M}}_{\text{soil(DL)}}\times {\text{SOC}}_{\text{cont}}\right)/1000$$

where SOC_ESM_ is the SOC mass in the sample soil mass layer (Mg/ha), M_soil(DL)_ is the mass of soil in the depth layer (Mg/ha; Equation 2), SOC_cont_ is the concentration of organic C (kg/Mg) from Equation (1) and 1,000 is the conversion factor from kg/ha to Mg/ha.

The carbon content of 5 mg of untreated milled soil and 2 mg of below‐ground biomass was measured using an ECS 4010 (Costech Analytical Technologies Inc., CA) elemental analyser. Soil and below‐ground biomass *δ* ^13^C was measured using a Picarro Cavity Ringdown Spectrometer G2131‐i (Picarro Inc., CA) coupled to the ECS 4010 using a Picarro Caddy split‐flow interface (Balslev‐Clausen, Dahl, Saad, & Rosing, 2013), and cane (−11.64‰) and beet sugar (−26.03‰; Iso‐Analytical, Crewe, UK) were used as isotopic reference standards. *δ* ^13^C was defined by Equation (4):

(4) $$\delta^{13}\text{C}=\left(\left(\left({}^{13}\text{C}/{}^{12}\text{C}\right)/\left({}^{13}\text{C}/{}^{12}{\text{C}}_{\mathrm{PDB}}\right)\right)-1\right)\times 1000$$

where ^13^C/^12^C_PDB_ is the isotopic ratio of the Pee Dee Belemnite standard material (0.0112372) and ^13^C/^12^C is the isotopic ratio of the measured below‐ground biomass or soil sample.

*Miscanthus* contribution to soil carbon (C_mis_) at T_6_ and T_12_ was calculated using Equation (5):

(5) $${\text{C}}_{\text{mis}}=\left(\delta_{n}-\delta_{0}/\delta_{r}-\delta_{0}\right)$$

where *δ*_0_ is the soil carbon isotope abundance at T_0_, *δ_n_* is the abundance at T_6 _or T_12_ and *δ_r_* is the abundance of the below‐ground biomass at T_6_ or T_12_ (Balesdent, Mariotti, & Guillet, 1987).

### 2.4 Modelling

The ECOSSE model (Smith et al., 2010) was run from the conversion year in 2005 and projected to 2020 using the ‘limited data site simulation’ mode for a continued grassland scenario and a LUC from grassland to *Mxg* scenario.

A default water table depth of 3 m with drainage class 2 was used. Soil texture percentages were sand 58%, silt 24% and clay 18% with a soil pH of 6 (Zatta et al., 2014). Long‐term monthly averages for precipitation and air temperature as well as monthly 2005–2011 data were taken from the nearby (~0.7 km) Gogerddan weather station ([Link]). As data were not available from this station for the years 2012–2016, meteorological data to cover this period were taken from another station approximately ~3.5 km distance (McCalmont, McNamara, Donnison, Farrar, & Clifton‐Brown, 2017). Monthly potential evapotranspiration from 2005 to 2016 was calculated using data from both weather stations using the R (R Core Team, 2015) package ‘Evapotranspiration’ (Guo & Westra, 2016). Meteorological conditions from 2016 to 2020 were predicted by ECOSSE using the long‐term monthly averages.

For the continued grassland land use scenario, the values for initial carbon content (77 Mg C/ha) and bulk density (1.14 g/cm^3^ and 1.11 g/cm^3^ for the 0–15 and 15–30 cm depths, respectively) were taken from Zatta et al. (2014), along with a yearly plant yield of 8 Mg dry matter (DM)/ha based on average values for this area given in Smit, Metzger, and Ewert (2008).

For the grassland to *Mxg* LUC scenario, the initial carbon content (78.8 Mg C/ha) was based on the value in Zatta et al. (2014) which included inputs from the herbicide‐killed pasture. All other initial details for the grassland and *Mxg* land use remained the same with the exception of the bulk density under *Mxg* which was taken from T_6_ data (1.08 g/cm^3^ and 1.13 g/cm^3^ for the 0–15 and 15–30 cm depths respectively; Zatta et al., 2014).

Input of C to the soil from crop residue and below‐ground biomass is calculated by ECOSSE as a function of net primary production (NPP) modified by empirical parameters within the model relating to each plant type (e.g. to account for harvest offtake). Further details can be found in Smith et al. (2010) and Dondini, Richards, Pogson, McCalmont et al. (2016b). Briefly, plant inputs enter the soil as a resistant plant material (RPM) and as a decomposable plant material (DPM) with a DPM:RPM ratio set depending on land use category (e.g. grassland or *Miscanthus*). There are five pools of soil organic matter (SOM) that each decompose at a specific rate constant and are sensitive to soil and climate data. There are specific C and N cycles within the model for grassland and *Miscanthus*. Decomposition is simulated by a number of equations into either BIO (‘biomass’ or active organic matter) or HUM (‘humus’ or more slowly turning over soil organic matter) pools, with inert organic matter (IOM) not contributing to the decomposition processes. In LUC scenarios, protected SOM (soil organic matter) is released from HUM to DPM and RPM. For the LUC to *Mxg* scenario, NPP (Table 1) was calculated from the spring‐harvested yield (P. Robson & J. Clifton‐Brown, unpublished data) plus 33% to account for over‐winter ripening loss (primarily leaf litter drop, based on the relationship outlined in Clifton‐Brown et al., 2007) and 20% to account for below‐ground biomass gain (estimated from the weight of oven‐dried coarse roots and rhizomes sampled over a 4 year period from a nearby established *Mxg* plantation (J.P. McCalmont, unpublished data). As in Zatta et al. (2014), for the conversion year, 1.5 Mg DM/ha was added to account for the input from the herbicide‐sprayed pasture and an estimated NPP of 16 Mg DM/ha (approximate mean NPP for years 11 and 12) was used for the projected growing seasons (2017–2020), when yields are expected to reduce towards the end of the commercial crop lifespan (Clifton‐Brown et al., 2015; Larsen, Jørgensen, Kjeldsen, & Lærke, 2014).

**Table 1:** Estimated net primary production (NPP) of biomass (as dry matter [DM]) calculated from the peak yield plus 20% as an approximation of below biomass gain for the land‐use change (LUC) from grassland to *Miscanthus x giganteus* scenario

Growing season	NPP (Mg/ha)
2005	1.9
2006	2.2
2007	16.7
2008	23.2
2009	21.2
2010	22.0
2011	26.3
2012	22.9
2013	21.7
2014	18.3
2015	14.3
2016	19.3
2017–2020	16.0

The root mean square error (RMSE) and relative error (RE) were used to evaluate the accuracy of the model outcomes compared to estimates of SOC derived from soil cores at T_6_ and T_12_.

### 2.5 Global warming potential

The difference between the ECOSSE‐predicted grassland and *Mxg* SOC at the end of 2020 (15 years after LUC) was converted from Mg C/ha to Mg CO_2_‐eq/ha using the molecular weight (IPCC, 2007). This was converted to a GWP (g CO_2_‐eq/MJ) using an estimated cumulative yield for a 15 year period of 180 Mg DM/ha (Larsen et al., 2014) and an energy content of 17.95 GJ/Mg DM (Felten, Fröba, Fries, & Emmerling, 2013). This GWP, relating to the difference in SOC, is compared and added to a previously published LCA value for *Miscanthus* cultivation, 4.4 g CO_2_‐eq/MJ (Hastings et al., 2017), that excluded changes in SOC stocks but included the entire supply chain (propagation, harvest, pelleting and transport) with a *Miscanthus* higher heating value of 18 GJ/Mg DM (Collura, Azambre, Finqueneisel, Zimny, & Weber, 2006; Hastings et al., 2017).

To consider the inclusion of other GHG costs relating to the LUC, the carbon cost of increased soil N_2_O emissions over the establishment to *Miscanthus* (4.13 Mg CO_2_‐eq/ha [8.83 kg N_2_O‐N/ha], Holder et al., 2019), and reversion process back to grassland (3.41 Mg CO_2_‐eq/ha [7.29 kg N_2_O‐N/ha], McCalmont et al., 2018), were converted to g CO_2_‐eq/MJ using the cumulative 15 year yield. In both N_2_O studies, no fertilizer was used during the *Miscanthus* management or LUC, and emissions were estimated from weekly (over a 20 month period, McCalmont et al., 2018) or biweekly (over an 18 month period, Holder et al., 2019) static chamber sampling.

### 2.6 Data analysis

Data analysis was performed in R version 3.5.1 (R Core Team, 2015), and model assumptions were tested using the Levene's and Shapiro–Wilk tests. At T_0_, the mean of the five soil core samples per plot was used to provide one value for each plot sampled. At T_6_ and T_12_, the three cores samples per plot were scaled (as detailed in the methods) and added together to give one value per plot.

To assess the effect of LUC on soil carbon stock, mean block level T_0_ SOC was compared to mean block level T_6_ and T_12_ SOC using a linear mixed‐effect model from package ‘nlme’ (Pinheiro et al., 2017) with time point as the fixed factor (T_0_, T_6_, T_12_), the random effect of block and an autocorrelation structure (AR1). Data were subsequently split into two groups (T_0_ with T_6_, and T_0 _with T_12_) to allow the influence of hybrid on changes in total scaled SOC stock compared to pre‐conversion values (T_0_). Land use (*Mxg*, Hyb 1–4 and pre‐conversion grassland) was used as a fixed factor with the random effect of block. Finally, T_6_ and T_12 _data were grouped to test the impacts on SOC stocks of the fixed factors: time point, hybrid, and depth and their interactions, with block included as a random factor. Model results were summarized using type III ANOVA (package ‘car’, Fox & Weisberg, 2011) and Tukey HSD (package ‘multcomp’, Hothorn, Bretz, & Westfall, 2008) post hoc tests.

*Miscanthus* C percentage contribution (C_mis_) data were split into 0–15 cm and 15–30 cm depths. Data for the 15–30 cm depth were log transformed to improve residuals. The contribution of C_mis_ to the total SOC stock was then explored with the hybrid, time points (T_6_, T_12_) and sampling positions (C_i_, C_e_, C_c_) included as fixed factors with the random effect of block.

Below‐ground biomass for each depth and sample position was analysed separately using non‐parametric paired Wilcoxon tests as residuals were not significantly improved using transformations. Correlations between SOC and C_mis_ versus below‐ground biomass, and SOC versus ripening loss were completed using the linear model function.

## 3 RESULTS

### 3.1 Soil organic carbon

**Figure 1:**
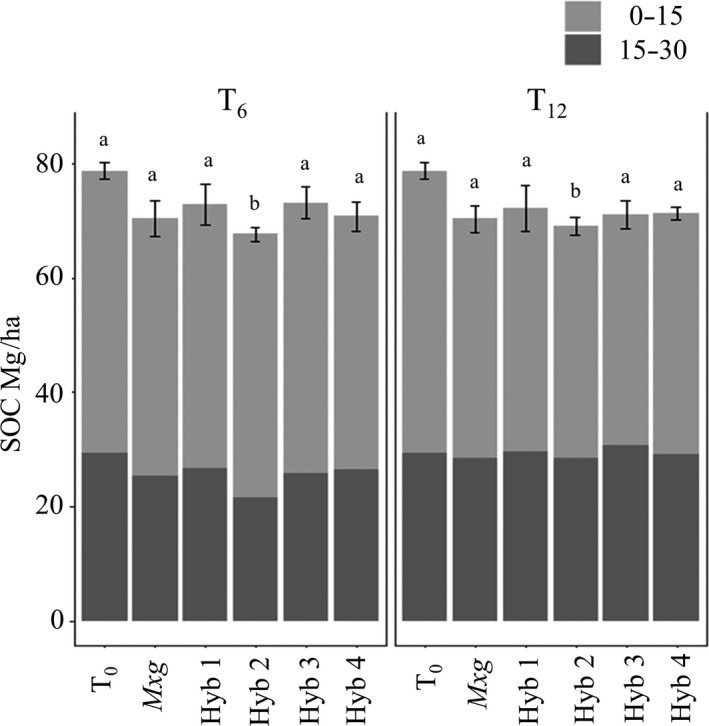
Soil organic carbon (SOC) in 0–15 and 15–30 cm depths, pre‐conversion (T_0_) from grassland to *Miscanthus x giganteus* (*Mxg*) and four *Miscanthus* hybrids (Hyb 1–4), 6 years after conversion (T_6_) and 12 years after conversion (T_12_). Error bars show the standard error of the mean for the total 0–30 cm values, and the same letter indicates non‐significant difference (*p* > 0.05)

Mean SOC (0–30 cm depth) at T_12_ was 71 ± 1 (standard error [SE]) Mg/ha, (SOC_ESM_ 67 ± 1 (SE) Mg/ha, for a reference soil mass layer of 0–3,000 Mg/ha). Soil bulk density results for each time point are summarized in Table 2. SOC was effected by year (χ^2^ (2) = 16.52, *p* < 0.001) with post hoc testing showing that both T_6_ and T_12_ were significantly lower than T_0_ (79 ± 1 (SE) Mg/ha), but that T_12_ was not significantly different to T_6_ (71 ± 1 (SE) Mg/ha). However, in subsequent analysis by hybrid, the difference to T_0_ is only significant (*p* < 0.05) for Hyb 2 (Figure 1). Between T_6_ and T_12_ SOC, both reduced in 0–15 cm layer and increased in the 15–30 cm layer by 4 Mg/ha (χ^2^ (1) = 18.08, *p* < 0.0001).

**Table 2:** Soil bulk density for the two soil depths at each sampling occasion (T_0_ and T_6_ from Zatta et al., 2014)

Depth (cm)	T_0_	T_6_	T_12_
0–15	1.14	1.08	1.04
15–30	1.11	1.13	1.21

**Figure 2:**
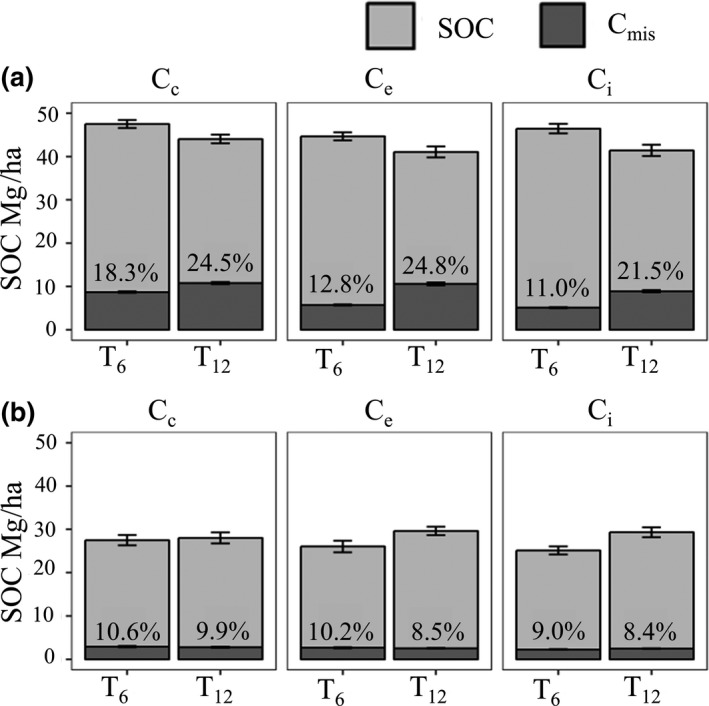
Total soil organic carbon (SOC) and *Miscanthus*‐derived carbon (C_mis_) after 6 (T_6_) and 12 (T_12_) years at each sample position (plant centre (C_c_), plant edge (C_e_) and inter‐row (C_i_)) for (a) 0–15 cm depth and (b) 15–30 cm depth. Percentages shown are the C_mis_ portion of SOC. Error bars show the standard error for separate C_mis_‐ and C_3_‐derived carbon

*Miscanthus* contribution (C_mis_) to SOC in the 0–15 cm layer (Figure 2a) was effected by sample position (χ^2^ (2) = 19.78, *p* < 0.001) decreasing with distance from the plant centre. However, at T_12_, C_mis_ was spread out more evenly across the three sampling positions than at T_6_ (χ^2^ (2) = 8.08, *p* = 0.02). In contrast, in the 15–30 cm layer, C_mis_ was similar in all positions (Figure 2b), although it decreased with Hyb 2 and Hyb 4 by 2% (χ^2^ (4) = 22.36, *p* < 0.001).

### 3.2 Biomass

**Figure 3:**
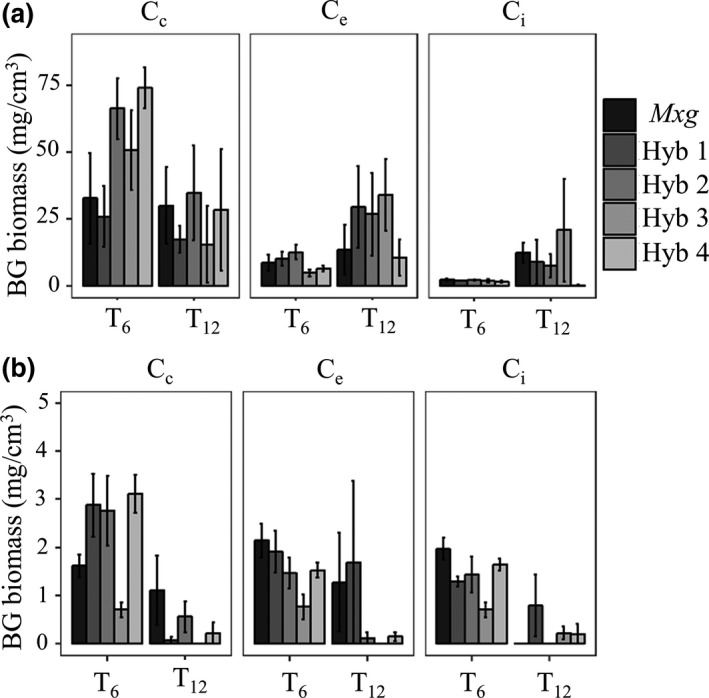
Mean below‐ground (BG) biomass (roots and rhizomes) found after 6 (T_6_) and 12 (T_12_) years of growth for *Miscanthus* hybrids (*Miscanthus x giganteus* (*Mxg*) and Hyb 1–4) at each sample position (plant centre [C_c_], plant edge [C_e_] and inter‐row [C_i_]) at the (a) 0–15 cm depth and (b) 15–30 cm depth. Error bars show the standard error

The distribution of below‐ground biomass (roots and rhizome) also changed from T_6_ to T_12_ with outward spread from the original planting position towards the inter‐row in the upper soil depth (Figure 3).

At the 0–15 cm depth, below‐ground biomass was only reduced at position C_c_ (*p* = 0.02) between time points T_6 _and T_12_ (by 37 ± 10 (SE) Mg/ha), whereas there was a reduction in all positions in the lower 15–30 cm layer (*p* < 0.05; Figure 3).

**Figure 4:**
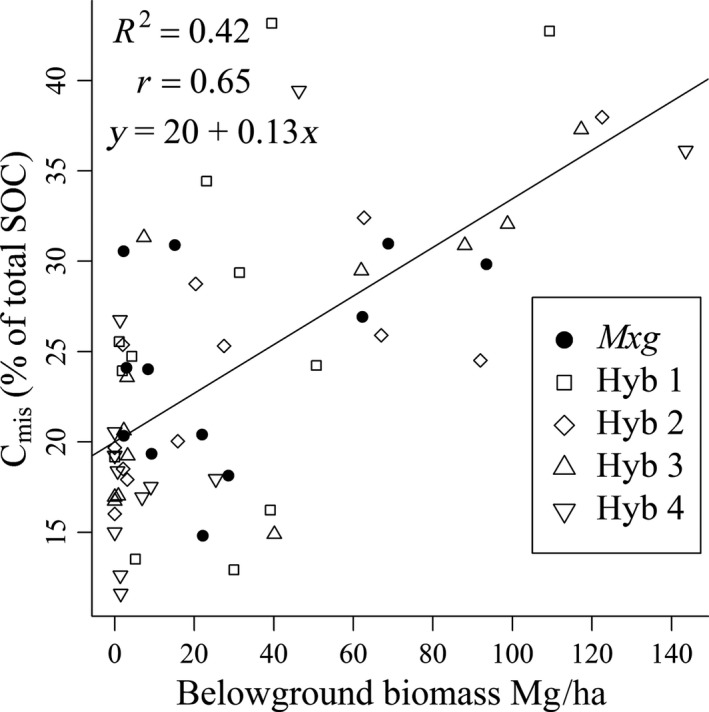
*Miscanthus*‐derived soil carbon as a percentage of total soil organic carbon (SOC) against below‐ground biomass for hybrids *Miscanthus x giganteus* (*Mxg*) and Hyb 1–4. Data includes all sample positions in the 0–15 cm soil layer at 12 years after planting

No correlation was found between below‐ground biomass and SOC at T_12_ as was found in T_6_ (Zatta et al., 2014). However, C_mis_ was positively and significantly correlated with below‐ground biomass at both time points (*r* = 0.67 at T_6_; and *r* = 0.65, *p* < 0.0001 at T_12_) in the upper 0–15 cm soil depth (Figure 4). Roots were not separated from rhizome in T_6_ or T_12_ but only small fragments of rhizome were found in samples from the lower depth at both time points.

Hyb 4 had the greatest reduction in below‐ground biomass in the 0–15 cm soil depth between time points (−14 ± 12 mg/cm^3^, T_6_ to T_12_) and also had the highest percentage inputs from ripening losses (leaf/litter drop; 36%, Table 3).

**Table 3:** Change in below‐ground (BG) biomass and *Miscanthus*‐derived soil carbon (as a percentage of total soil organic carbon [SOC]) at 0–15 cm depth after 6 (T_6_) and 12 (T_12_) years of land conversion from grassland to *Miscanthus*. Biomass and C_mis_ differences are taken from mean values across all three sampling positions (C_c_, C_e_, C_i_). Above‐ground ripening loss is the difference between autumn peak and spring harvest yields. The standard error is shown in brackets

Hybrid	BG biomass (mg/cm^3^): Difference T_6_ to T_12_	C_mis_ (% of SOC): Difference T_6_ to T_12_	Above‐ground ripening loss (%)
*Mxg*	+4 (±10)	+10 (±2)	26 (±9)
Hyb 1	+6 (±6)	+10 (±3)	31 (±4)
Hyb 2	−4 (±9)	+5 (±1)	19 (±1)
Hyb 3	+4 (±12)	+8 (±3)	25 (±8)
Hyb 4	−14 (±12)	+7 (±3)	36 (±4)

**Figure 5:**
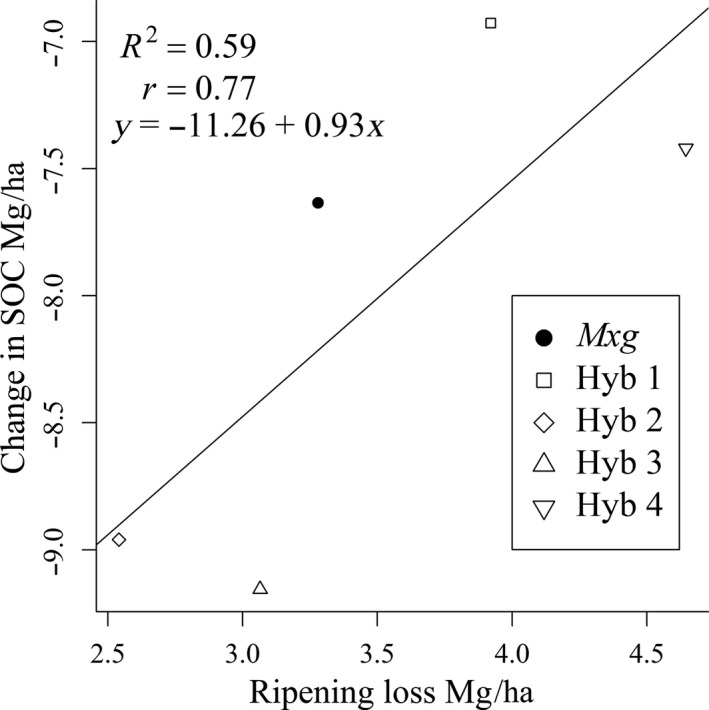
Correlation between change in T_0_ and T_12_ mean soil organic carbon (SOC) and estimated ripening loss at the 0–15 cm depth for hybrids *Miscanthus x giganteus* (*Mxg*) and Hyb 1–4

Hyb 2 had the lowest over‐winter ripening loss although no significant difference was found between ripening loss for the different *Miscanthus* hybrids. Ripening loss was positively, but not significantly, correlated with change in SOC (between T_0_ and T_12_) in the 0–15 cm depth layer (*r* = 0.77, *p* = 0.13, Figure 5).

### 3.3 Modelling

Measured SOC at T_6_ and T_12_ was within the 95% confidence interval (CI) of the ECOSSE model predictions for all the hybrids. For the LUC from grassland to *Mxg* scenario, the model RMSE of 5.49% was within the RMSE 95% CI of 9.67%, and the RE of 5.41% was within the RE 95% CI of 9.62% (based on soil core results from T_6_ and T_12_).

**Figure 6:**
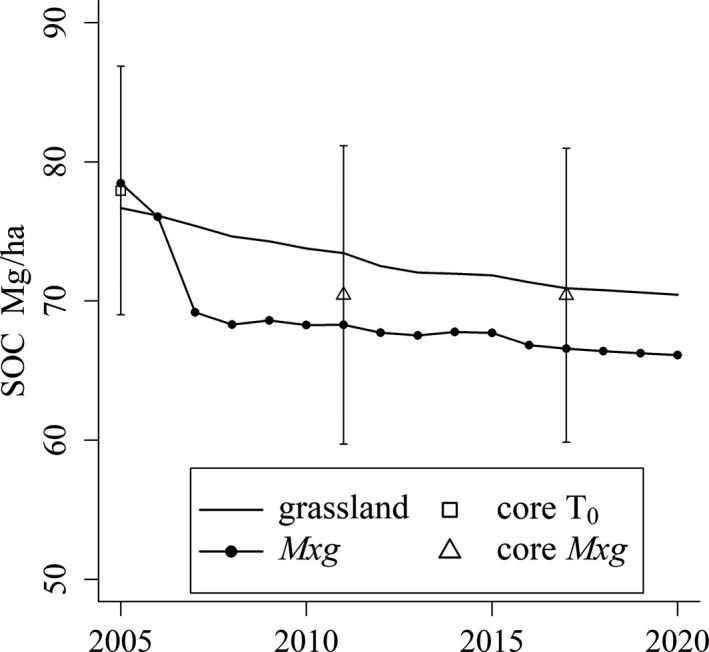
Results of the 15 year (2005–2020) ECOSSE simulation of soil organic carbon (SOC) under a continued grassland scenario (grassland) and a land‐use change (LUC) from grassland to *Miscanthus x giganteus* (*Mxg*) scenario. Mean SOC from soil cores taken immediately pre‐conversion (T_0_) and from under *Mxg* in 2011 and 2017 are shown with error bars indicating the 95% confidence intervals

At the beginning of the 15 year simulation, the LUC to *Mxg* scenario shows slightly higher SOC than the continued grassland scenario (reflecting the higher initial C value used). After this, there is a clear drop in levels of SOC under *Miscanthus* before they begin to level out. After 15 years, the predicted loss compared to T_0_ was 12 Mg/ha; however, the model suggests there is also a slow decline in the SOC under the continued grassland scenario which shows a loss of 7 Mg/ha after 15 years (Figure 6). At the end of 2020, the difference in SOC stocks between the continued grassland scenario, and LUC to *Mxg* scenario is 4 Mg C/ha.

### 3.4 Life cycle analysis

The carbon cost relating to the difference in predicted SOC stocks between the continued grassland and LUC to *Mxg* scenarios of 4 Mg C/ha (or 15 Mg CO_2_‐eq/ha) equates to 5 g CO_2_‐eq/MJ based on the energy content of the estimated 15 year yield. This represents a 125% increase when added to a previous LCA that excluded soil carbon changes (Table 4).

**Table 4:** Global warming potential (GWP) over a 15 year crop lifetime of the estimated carbon costs associated with the *Miscanthus* production chain, predicted difference in soil organic carbon (SOC) stocks (compared to a grassland counterfactual), and estimated increases in soil nitrous oxide (N_2_O) emissions related to the land conversion and reversion

Cost association	GWP (g CO_2_‐eq)	GWP (sum, g CO_2_‐eq)
Production chain (Hastings et al., 2017)	4	
Difference in SOC	5	9
Establishment N_2_O (Holder et al., 2019)	1	10
Reversion N_2_O (McCalmont et al., 2018)	1	11

## 4 DISCUSSION

### 4.1 Total soil organic carbon

In the light of concerns over the impact on soil carbon when planting bioenergy crops into grassland (McCalmont, Hastings, et al., 2017; Whitaker et al., 2018), this study has shown a 10% loss in SOC after 12 years of LUC change from this temperate marginal grassland to *Miscanthus* at this site. In this new analysis, unlike Zatta et al. (2014), we did find a reduction in soil carbon stock at T_6_ compared to T_0_ but the breakdown by hybrid confirmed that the difference was only significant for a single hybrid (at T_6_ and T_12_, Figure 1). The overall reduction in carbon from T_0_ to T_12_, of 8 Mg/ha, is within the range +4 to −9 Mg/ha reported in other grassland to *Miscanthus* field‐based studies (Clifton‐Brown et al., 2007; Schneckenberger & Kuzyakov, 2007; Zang et al., 2018; Zimmermann et al., 2012). There was also no difference between carbon stocks at the two sampling points (T_6_ and T_12_) suggesting a reasonably stable carbon state. However, this is in contrast to Zang et al. (2018) where soil organic matter increased between sampling occasions (9 and 21 years after *Miscanthus* planting). This difference may be as a result of different soil pH and nutrient levels, or the slightly cooler (annual average air temperature 6.7°C vs. 10.4°C) and wetter (annual average precipitation 1,074 mm vs. 654 mm) climate in this study, which could all influence *Miscanthus*‐derived carbon (Zimmermann et al., 2012).

The initial tillage and planting of the cover crop in this study occurred in the autumn (October 2004) before the T_0_ samples were taken in early May 2005 (prior to *Miscanthus* planting). It is therefore possible that if the original sampling had taken place in the autumn, estimated SOC stock may have been higher. Tillage results in releases of SOC due to the change in conditions that are created in the soil matrix and the creation of newly available substrate that can stimulate soil bacteria/microbial activity and decomposition rates. However, initial increases in CO_2_ immediately following autumn ploughing have mainly been attributed to the release of soil CO_2_ from large soil pores and from the release of dissolved CO_2_ from soil water, and there is generally a lag time before CO_2_ from bacterial decomposition of soil organic matter and SOC is released (Reicosky & Lindstrom, 1995). Turnover times for light fraction SOC are generally in terms of months to years (Post & Kwon, 2000) and are connected to soil moisture and temperature, with temperature increases stimulating turnover (La Scala Jr. et al., 2008). During the winter months following tillage at this experimental site, microbial activity and decomposition could be expected to be slow, due to low air and soil temperatures (mean air temperature at the site October 2004–April 2005 was 8°C) and therefore changes in SOC from October to April minimal. Baseline soil carbon stocks at our site were also remarkably similar to another nearby periodically re‐seeded grassland site used for a land use transition experiment (see McCalmont, McNamara, et al., 2017), which contained 79 Mg C/ha in the top 30 cm. Results presented here might, therefore, be assumed to be reasonably representative of land use transitions on these typical improved marginal grassland systems in the United Kingdom. Grasslands with deeper soils have shown contrasting changes to SOC following LUC to *Miscanthus*. In empirical studies that sampled soils to a depth of 1 m across a range of soil types, Rowe et al. (2016) found that significant SOC losses were only found in the top 30 cm, whereas Qin et al. (2016) found SOC was generally increased in the top 30 cm. However, both studies conclude that taken over the whole 1 m soil profile SOC was not significantly lost. In some cases, surface losses were offset by increases lower in the profile and in others changes were limited to the surface and therefore impacts were diluted when considered over the whole depth. Impacts may also be different for longer term, semi‐natural grassland sites where initial carbon stocks may be higher (Guo & Gifford, 2002).

### 4.2 Miscanthus derived carbon and spatial distribution

C_mis_ mirrored the ground cover survey and below‐ground biomass found (with the spreading of *Miscanthus* into the outer C_e_ and C_i_ sampling positions) supporting the use of multiple coring positions when scaling up from small samples to Mg/ha (Neukirchen, Himken, Lammel, Czypionka‐Krause, & Olfs, 1999).

The land‐use change is clearly seen in the increase of C_mis_ between T_0_ and T_6_. Although new *Miscanthus* C_4_ carbon replaced pre‐existing C_3_ carbon, this was not at a high enough rate to completely offset losses by the end of year 12. The impact of LUC on SOC generally differs with soil depth (Poeplau & Don, 2014; Rowe et al., 2016; Zang et al., 2018). In this study, it was found that between T_6_ and T_12_ C_mis_ increased in the top layer, although SOC also declined (Figure 2). A higher percentage of C_mis_ in the topsoil (0–10 cm) compared to deeper soil layers is in accordance with findings by Poeplau and Don, (2014) and Hu, Schäfer, Duplay, and Kuhn (2018). This is likely to be attributed to the distribution of the main *Miscanthus* root and rhizome biomass, which are concentrated in the upper layer (Figure 3) and positively correlated to C_mis_ at T_6_ and T_12_ (Figure 4). However, SOC also declined in this upper layer, which may be in part attributed to the ‘priming effect’ where increased microbial activity (stimulated by ploughing and an increase in accessible C generated from higher plant biomass, root exudates and litter) leads to the use of more stable soil carbon (Cheng, 2009; Hopkins et al., 2013; Kuzyakov, 2010). In contrast, between T_6_ and T_12_, SOC in the lower 15–30 cm depth increased despite C_mis_ remaining at a similar level (Figure 2). The reason for this difference is unclear, but it may be a legacy of the cultivation where although ploughing could be expected to add C_3_ inputs from dead roots/residues in both soil depths there are slower turnover rates at the lower 15–30 cm layer due to the higher bulk density (Table 2) resulting in less aeration for microbial activity. The increase in SOC in this lower layer was only seen at the plant edge and inter‐row positions where there is also the increased possibility of weeds providing C_3_ inputs to the soil, but further research would be needed to confirm these possibilities.

### 4.3 Influence of hybrid

Despite the novel hybrids (Hyb 1–4) having lower lignin content than *Mxg*, and three out of the four novel hybrids having a lower C:N ratio, the influence of hybrid was small. This is in contrast to the suggestion made in Zatta et al. (2014) that after a longer time period differences in the SOC levels for the hybrids would reflect differences in carbon partitioning. All five hybrids sequestered similar amounts of C_mis_ and only Hyb 2 had lower overall SOC compared to the baseline (at T_6_ and T_12_). Therefore, this study suggests that for this type of interspecies hybrid (*M. sacchariflorus x M. sinensis*) the potential of yield improvements are not generally at the cost of soil carbon losses compared to the commercial standard *Mxg*. However, investigation into differences in the chemical and physical properties of the root biomass of Hyb 2 may provide more insights.

Leaf litter inputs to the soil are an important part of carbon cycling (Amougou et al., 2011) and we found that Hyb 4, which lost the most below‐ground biomass between T_6_ and T_12_, also had the highest ripening loss which may have acted as compensation. Hyb 2, the only hybrid with significantly lower SOC than at T_0_, also had low ripening loss inputs (Table 3). The correlation between ripening loss and change in SOC found in this study after 12 years (Figure 5), although not significant is in line with the prediction from the RothC model in Zatta et al. (2014) where ripening loss for each hybrid was correlated to projected SOC in 2025.

### 4.4 Modelling

The ECOSSE model predicted SOC under *Miscanthus* within the statistical error of the field measurements and no bias was found. However, SOC under *Mxg* projected to 2020 with ECOSSE (66 Mg C/ha) is less than was predicted using the RothC model (72 Mg C/ha, Zatta et al., 2014). The initial drop in SOC following land use conversion to *Mxg* is greater with ECOSSE, which may be attributed to the LUC routine within ECOSSE which aims to simulate carbon loss from cultivation. Differences in predictions may also be as a result of differences in weather data used in the two models after 2011. However, both models predicted the SOC to within the 95% CIs at T_6_ and T_12_ when soil core samples were taken. Although the model can be run using different yield results for the novel hybrids, differences in decomposition rates for above‐ and below‐ground biomass would allow for greater accuracy in comparisons of genotypic differences.

In this work, it was not possible to compare samples from maintained grassland at the same site or within an acceptable distance but the ECOSSE model suggests SOC under continued grassland also has a steady decline of 7 Mg/ha over 15 years (Figure 6). It should not therefore be assumed that even without any cultivation (whether to *Miscanthus* or a new grass ley) SOC would remain the same as baseline levels over time. UK wide surveys recording trends in soil carbon over time (at the 0–15 cm depth) have also reported significant reductions (~6%) in soil carbon under managed fertile grasslands between 1998 and 2007 (Bellamy, Loveland, Bradley, Lark, & Kirk, 2005; Emmett et al., 2010). These losses may be attributable to a number of factors including climate change and changes in management methods resulting in more efficient harvesting and a reduced use of organic manures (Bellamy et al., 2005; Smith et al., 2007). The grassland scenario is run with the same yearly biomass yield input, whereas changes in weather and management would in reality impact on yields, and hence carbon inputs, resulting in differences in SOC.

The difference in predicted SOC between the LUC change and continued grassland scenarios (−6%, at 2020, the end of the estimated *Mxg* crop lifetime) was within the range of −48% to +15% found for eight established (>5 years) *Miscanthus* plantations compared to neighbouring grassland sites (Rowe et al., 2016). The contrasting results for the different sites within Rowe et al. (2016), along with the results of this study, show that significant losses in SOC can occur, and while Qin et al., (2016) found no overall change in SOC in relation to grassland to *Miscanthus* conversions, CIs ranged from −9% to +21% (for the mean of five datasets reflecting the change in SOC in *Miscanthus* crops >10 years).

### 4.5 Global warming potential impacts

Soil sustainability is an important consideration when assessing the impacts of potential LUC scenarios ([Link]; Hillier et al., 2009). In this long‐term LUC study where initial SOC stocks are similar to that expected for temperate grasslands in this climate ([Link]; Kiely et al., 2009; McCalmont, McNamara, et al., 2017), we have seen decreases in SOC (compared to baseline levels, and between modelled predictions of grassland and *Miscanthus*), which more than doubled a production cost LCA result (Table 4). Similarly soil N_2_O emissions during crop establishment and reversion to the next crop have recently been shown to represent a significant portion of the greenhouse gas balance (Holder et al., 2019; McCalmont et al., 2018).

The starting *Miscanthus* production GWP figure used of 4 g CO_2_‐eq/MJ from Hastings et al. (2017) does not include changes in soil carbon stocks or soil greenhouse gas fluxes, based on the premise that on average C would be sequestered or at worst maintained. However, when the cost of change in soil carbon (4 Mg C/ha, 5 g CO_2_‐eq/MJ, compared to a continued grassland counterfactual), along with the cost of soil N_2_O emissions from land conversion (1 g CO_2_‐eq/MJ, Holder et al., 2019) were added to the original GWP, the resulting cost of producing a *Miscanthus* crop over a 15 year period (10 g CO_2_‐eq/MJ or 180 kg CO_2_‐eq/Mg DM) still remained far lower than estimates for producing energy from natural gas (59 g CO_2_‐eq/MJ), currently the highest consumed fossil fuel energy source (BEIS, 2018), and coal (121 g CO_2_‐eq/MJ; Hastings et al., 2017).

Whether the bioenergy crop itself should bear the greenhouse gas cost of land conversion at the beginning of the cropping cycle (Holder et al., 2019) or reversion at the end (McCalmont et al., 2018), or indeed both, is open to debate. It may also be the case that any losses in SOC are temporary depending on the LUC after *Miscanthus*, if for example the land is re‐converted to a permanent pasture. As shown in McCalmont et al. (2018) soil N_2_O emissions connected to cultivation disturbances are strongly driven by the legacy of the previous crop species, and losses or gains in soil carbon are also sensitive to the initial land condition (Qin et al., 2016; Richards et al., 2017) suggesting a case for LCA studies to attribute conversion period greenhouse gas emissions to the previous crop and incorporate projected reversion costs into the GWP balance of the current one.
