# Supplementary material for: Measured and modelled effect of land‐use change from temperate grassland to Miscanthus on soil carbon stocks after 12 years
Source: Glob Change Biol Bioenergy. 2019 May 21;11(10):1173–86. doi: 10.1111/gcbb.12624 (PMC6774323; doi:10.1111/gcbb.12624)
Supplement: Supplementary file 1 [file GCBB-11-1173-s001.docx]

**Supplementary Information**

**S1** *Determining the percentage area covered by each core sample position.*

The same soil core sampling design was used in T_12_ (12 years after planting) as previously used in T_6_ (six years after planting, Zatta *et al*., 2014). The design uses three soil cores taken from each plot to represent the areas covered by the plant centre (C_c_), plant edge (C_e_), and inter-row (C_i_). For this study (T_12_) the percentage area represented by each core location was determined by a field cover survey using three randomly placed 1 m^2^ quadrats per plot. The survey was conducted after the spring 2017 harvest when the remaining stubble was used to give an above ground indication of the area covered by the *Miscanthus* plants. The results showed that the *Miscanthus* plants covered a mean area of 63.50% +/-1.96 of each plot (Table S1).

**Table S1** Results of the ground cover survey to determine the percentage area covered by *Miscanthus*. The percentage cover and standard error (SE) shown are the mean of the three 1 m^2^ quadrats used per plot.

The distances between the three soil core locations within each plot were based on the original planting position, as it was not always possible to determine the location of individual plant edges due to the spreading nature of the mature plants. The soil core sample at C_c_ was taken at the point the plant was originally planted, C_i_ at 0.5 m along a diagonal between two plants (being the furthest distance), and C_e_ halfway between C_c_ and C_i_ (Fig. S1).


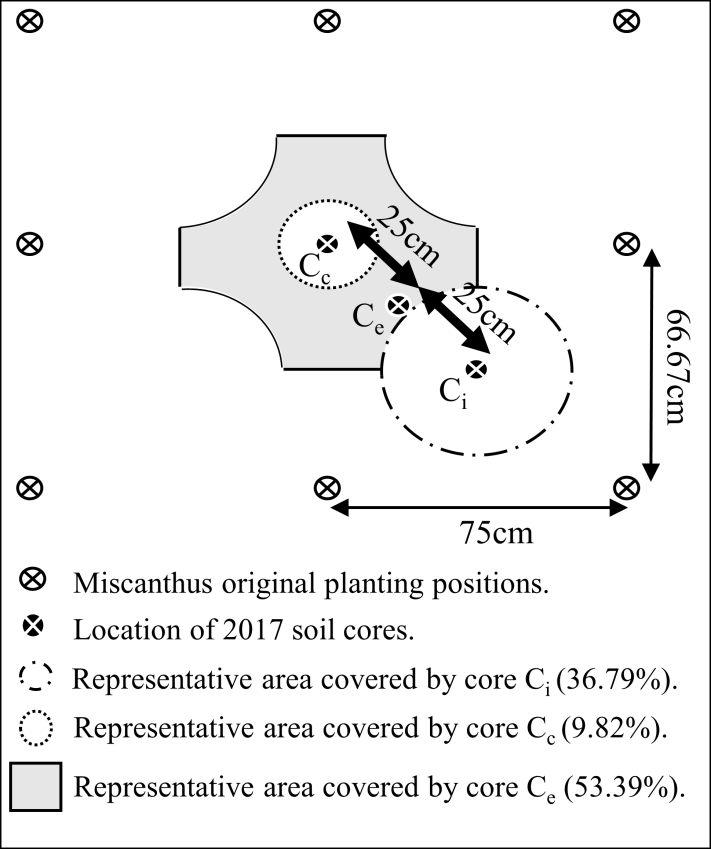


Fig. S1 Location of the three soil core positions taken within each plot, with the percentage area represented by the plant centre (C_c_), plant edge (C_e_) and inter-row (C_i_).

Based on the original planting distance the zone relating to an individual plant was 0.50 m^2^ (0.67 m x 0.75 m). The representative area covered by C_i_ of 0.18 m^2^ (37% of the zone), was taken from the field survey as the area without *Miscanthus* stubble. The remaining 63% (covered by *Miscanthus*) was split as follows: the area represented by C_c_ was given an arbitrary diameter of 0.25 m (area 0.05 m^2^) covering 10%; the remaining area of 0.27 m^2^ (53%) was taken to represent C_e_.

**S2** *Plot heterogeneity*

To explore heterogeneity between individual plots, the “aov” function in the statistical program R (R Core Team, 2015) was used to check for differences in soil organic carbon (SOC) between individual plots at time points T_0_, T_6_, T_12_ and also for the change in SOC between T_6_ and T_12_. No statistical differences were found (p>0.05).

**References**

R Core Team. (2015). R: A language and environment for statistical computing. R Foundation for Statistical Computing, Vienna, Austria. URL https://www.R-project.org/.

Zatta, A., Clifton-Brown, J., Robson, P., Hastings, A., & Monti, A. (2014). Land use change from C3 grassland to C4 Miscanthus: Effects on soil carbon content and estimated mitigation benefit after six years. *GCB Bioenergy*, *6*(4), 360–370. https://doi.org/10.1111/gcbb.12054
